# Measuring Spatial Accessibility of Urban Medical Facilities: A Case Study in Changning District of Shanghai in China

**DOI:** 10.3390/ijerph18189598

**Published:** 2021-09-12

**Authors:** Min Cheng, Li Tao, Yuejiao Lian, Weiwei Huang

**Affiliations:** School of Management, Shanghai University, 333 Nanchen Road, Baoshan District, Shanghai 200444, China; chengmin@shu.edu.cn (M.C.); lianyj_ly@126.com (Y.L.); Quiteww27@163.com (W.H.)

**Keywords:** medical facilities, spatial accessibility, improved potential model (IPM), geographic information systems (GIS), shanghai

## Abstract

Medical facilities help to ensure a higher quality of life and improve social welfare. The spatial accessibility determines the allocation fairness and efficiency of medical facilities. It also provides information about medical services that residents can share. Although critical, scholars often overlooked the level of medical facilities, the composition of integrated transportation networks, and the size of service catchment in the literature on accessibility. This study aims to fill this research gap by considering the integrated transportation network, population scale, travel impedance between medical facilities and residential areas, and the impact of medical facilities’ levels on residents’ medical choices. An improved potential model was constructed to analyze the spatial accessibility of medical facilities in Changning District of Shanghai, China. Interpolation analysis was conducted to reveal the spatial accessibility pattern. Cluster and outlier analysis and Getis-Ord G_i_* analysis were applied for the cluster analysis. Results show that the spatial accessibility of medical facilities is quite different in different residential areas of Changning District, Shanghai. Among them, the spatial accessibility of medical facilities is relatively high in Hongqiao subdistrict, Xinjing Town, and part of Xinhua Road subdistrict. In addition, residents have overall better access to secondary hospitals than to primary and tertiary hospitals in the study area. This study provides a spatial decision support system for urban planners and policymakers regarding improving the accessibility of healthcare facilities. It extends the literature on spatial planning of public facilities and could facilitate scientific decision making.

## 1. Introduction

Medical services are fundamentally significant to the public and closely related to people’s health and survival. As the basis of peoples’ health and life safety, medical facilities provide necessary medical services and resources for the public. Poor accessibility to medical facilities can affect individuals in many ways and lead to health problems not being addressed in time. A prior study proved that space barriers between patients and providers reduce healthcare utilization and decrease the use of preventive services [1]. The rational distribution of medical facilities guarantees the equal accessibility of the public to enjoy essential medical treatments, thereby eliminating spatial polarization and reducing spatial differentiation, which is significant to urban development [2,3,4]. It also determines the level of medical services in a city. An accessible medical facility layout is crucial for service quality and the public’s satisfaction [5]. Thus, investigating the distribution and availability of medical facilities and the rationality is important.

### 1.1. Potential Spatial Accessibility

Accessibility is a measure of the public healthcare service effectiveness and the medical resource allocation fairness [6]. Accessibility was defined as the opportunities for interaction and a desire and ability to overcome space separation [7]. Accessibility reflects the degree of matching between residents and medical facilities, which involves five dimensions, i.e., affordability, availability, accommodation, acceptability, and accessibility [8], two stages (potential and revealed), and spatial and non-spatial aspects [9].

Potential accessibility considers the possible use of public facilities for a given population and revealed that accessibility focuses on the actual utilization of public facilities. Spatial accessibility explores the significance of spatial separation between supply and demand as an obstacle or promoters, and non-spatial accessibility concerns the non-geographic barriers or promoters. In sum, accessibility has four types, i.e., potential spatial accessibility, potential non-spatial accessibility, revealed spatial accessibility, and revealed non-spatial accessibility. This study focuses on potential spatial accessibility, which depicts how easy it is to access medical facilities by overcoming travel impedance, depending on the location and distribution of medical facilities and traffic conditions.

### 1.2. Measuring Accessibility of Medical Facilities

To allocate medical resources rationally, planners should identify the areas and communities where service provision is inadequate. Scientifically measuring the accessibility of medical facilities helps to decide whether the layout is reasonable and provides crucial information for optimizing the configuration and public policy formulation. Scholars proposed several methods to evaluate the spatial accessibility to medical facilities. The most commonly used measurement includes population-to-provider ratio, distance to the nearest provider [10], kernel density method [9], the two-step floating catchment area (2SFCA) method [11,12], and the potential model (or gravity model) [7,13].

The population-to-provider ratio is the ratio of healthcare capacity to the population within an administrative area and is thereby easy to calculate and interpret. However, this method only reflects the ratio itself and neglects other factors, such as distances, which leads to inaccurate measurements. It also has received criticisms for its two assumptions: (1) the measurement limits residents into one administrative area and does not consider boundary-crossing. (2) The measurement does not incorporate distance or travel impedance. Residents can bypass the nearest medical facilities when multiple services are available.

As to the distance to the nearest provider method, it ignores the availability of supply. The kernel density method takes cross-border seeking for healthcare services and distance decay effect into account, and thus it is superior to the population-to-provider ratio method [9]. However, it has weaknesses in measuring population density and areas of medical services [14]. The 2SFCA method calculates the number of accessible facilities for a particular location by setting a threshold distance or travel time. It is difficult to determine the threshold distance or time, especially when there are different levels of medical facilities [13].

Compared with other accessibility methods, the potential model (or gravity model) reflects spatial competitions between facility suppliers and demanders, including the competition between facility suppliers for demanders and the competition between demanders for limited resources [4,9]. The potential model is complete in concept and more flexible in use than the above methods [15]. It follows Newton’s law of universal gravitation. It is a widely applied method in studying spatial interactions. This model supposes that residents’ spatial accessibility to medical services decreases with the increase of distance to nearby medical facilities; that is, the distance decay effects are considered. It also has advantages in capturing supply and demand characteristics.

### 1.3. Improvement of the Traditional Potential Model

The traditional potential model could date back to Hansen [7], who defined accessibility as the potential of opportunities for interactions. He considered all service facilities for the selection, which traded off the size or quality of healthcare facilities with travel impedance. In general, accessibility could improve with an increase in either the number of service supply points or the capacity at any supply points or when the distance traveled to medical facilities or the travel friction decreases. However, the traditional potential model only relates to the capacity of service facilities and travel impedance factors, without considering the population factor, i.e., the competition between the service demands for service resources provided by the same service facility. When the service facilities have the same service capabilities and travel impedance but cover different populations, the basic model cannot clearly distinguish their actual spatial accessibility. Accordingly, some scholars improved the traditional potential model by considering the population’s size [14,16].

Despite the above improvement, the improved potential model fails to consider the influence of facility levels. For medical facilities, the different levels of facilities (hospitals in China are divided into three tiers, i.e., primary, secondary, and tertiary, in accordance with their scale, service scope, function, task, and other characteristics) will affect the residents’ medical choice behavior. Thus, this study aims to fill this research gap.

### 1.4. Evaluating Spatial Accessibility

Studies often use geographic information systems (GIS) to perform complex geospatial computing tasks, e.g., conduct space planning for public service facilities [17]. One can identify underserved and poorly served areas by using GIS-based approaches (e.g., interpolation analysis and hot spot analysis). The results may help to achieve the spatial efficiency and equity of healthcare systems [18,19,20,21]. However, there are limitations with current studies.

Firstly, most of the existing studies assumed that people travel to medical facilities in a single transportation mode, in most cases, by car. Only a few studies compared the accessibility of different ways of travel. For example, Higgs et al. explored the effect of different travel methods on the correlation between different measures of general practitioner supply and area-level deprivation and the percentage of elderly patients [22]. The study area of this research is Changning District, Shanghai. Shanghai is an international metropolis and one of the most developed cities in China. It has a developed transportation system consisting of various travel modes. Residents in different areas have different transportation options to reach medical facilities, resulting in different traffic impedances and transit times. Due to complex transportation routes, this study considers the impact of the comprehensive transportation network composed of ground transportation and rail transit on accessibility for large cities.

Secondly, when using GIS for accessibility evaluation, studies usually employ counties (districts) or streets (towns) as the study units [4,23]. For medical facilities, calculating the spatial accessibility of small research units can more accurately reflect the rationality of allocating medical resources to judge the under-served residential areas more reasonably. Therefore, it is of significance to measure the spatial accessibility of medical facilities based on smaller units (i.e., community-scale).

To sum up, the objectives of this study are three-folded, including: (1) to assess and delineate the accessibility of medical facilities at the community level; (2) to identify areas with good/lousy accessibility to medical facilities, especially in dense population areas; and (3) to put forward suggestions for the government to optimize the configuration of medical facilities. The innovations of this study are also three-folded. First, it considers the comprehensive transportation network composed of ground transportation and rail transit. Second, it conducts community-scale analysis. Third, it improves the potential model by considering the influence of different levels of medical facilities on residents’ medical choices.

The remainder of this study is organized as follows. Section 2 introduces the methods used in this research, including improved potential model, interpolation analysis, and Getis-Ord G_i_* analysis. Section 3 introduces the study area and data sources. Section 4 presents the results, including the overall accessibility of medical facilities and the respective accessibility of different grades of medical facilities in the study area. Finally, Section 5 discusses our conclusions.

## 2. Methods

Given the advantages over other accessibility methods, the potential model was employed to calculate the spatial accessibility of residential areas to medical facilities of different levels. Improvements were made to the traditional potential model. Based on the output of improved potential model, spatial interpolation analysis was used to examine the spatial accessibility of medical facilities surrounding the measurement points.

**Figure 1:**
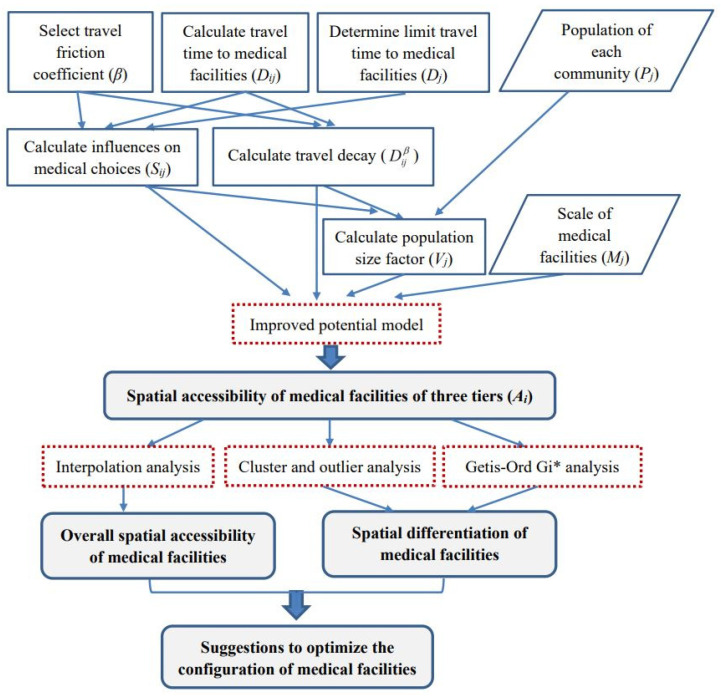
Methods workflow.

Further, cluster and outlier analysis and Getis-Ord G_i_* analysis were used to examine the spatial disparity of accessibility to medical facilities in the study area. The results of these two analyses were compared. Specifically, cluster and outlier analysis was conducted to identify spatial clusters with similar high or low values or spatial outliers with high/low values surrounded by low/high values. Getis-Ord G_i_* analysis was used to identify spatial clusters of statistically significant hot spots (clusters with high accessibility) and cold spots (clusters with low accessibility). In this way, areas with good/lousy accessibility to medical facilities of different levels can be identified. An overview of the methods employed in this study is illustrated in Figure 1.

### 2.1. Potential Model and Its Improvement

The potential model is a model that studies the interaction of economic and social space based on the physical law of gravity. It has been widely employed to measure the spatial accessibility of facilities with considering the spatial scale and distance decay. The traditional potential model is written as:

(1) $$A_{i}=\sum_{j=1}^{n}A_{ij}=\sum_{j=1}^{n}\frac{M_{j}}{D_{ij}^{\beta }}$$

where *A_j_* denotes the gravity of facility *j* to residential area *i* when the travel friction coefficient is *β,* which describes travel impedance; *A_ij_* is the total potential generated by all facilities; *M*_j_ is the scale of *j*; and *D_ij_* denotes the travel time, distance or generalized cost between *i* and *j*.

The basic potential model has failed to consider the effect of the size of the population radiated by the facility on accessibility. Joseph and Bantock introduced the population size factor *V_j_* and proposed an Improved Potential Model that considers demand competition as follows [24]:

(2) $$A_{i}=\sum_{j=1}^{n}\frac{S_{ij}M_{j}}{D_{ij}^{\beta }V_{j}},\;V_{j}=\sum_{k=1}^{m}\frac{S_{kj}P_{k}}{D_{kj}^{\beta }}$$

where *P*_k_ is the population of the residential area *k.*

The above expression explicitly considers the population distribution in the vicinity of various medical facilities. However, it does not consider the effect of different levels of medical facilities on residents’ medical choices. As mentioned before, hospitals are hierarchical. Since hospitals of different levels have different scales, technical levels, and service capabilities, their attractiveness to residents also varies. We set different limit travel times to reflect the influence of medical facilities level on the residents’ medical choices. Thus, the potential model can be further modified as follows:

(3) $$A_{i}=\sum_{j=1}^{n}\frac{S_{ij}M_{j}}{D_{ij}^{\beta }V_{j}},\;V_{j}=\sum_{k=1}^{m}\frac{S_{kj}P_{k}}{D_{kj}^{\beta }},\;S_{ij}=1-{\left(\frac{D_{ij}}{D_{j}}\right)}^{\beta }$$

where *S_ij_* is the influence coefficient of facility’s level; *S_kj_* represents the influence of the level of medical facility *j* on the medical choice of residents in residential area *k*; *D_j_* is the limit travel time (or distance) to facility *j*. When the travel impedance (travel time or distance) between residential area *I* and medical facility *j* is greater than the time or distance threshold (i.e., *D_ij_*/*D*_j_ ≥ 1), *S*_ij_ ≤ 0. In this case, the residents at the residential area *I* will be reluctant to seek medical treatment at medical facility *j*.

### 2.2. Interpolation Analysis

To examine the spatial accessibility of medical facilities surrounding the measurement points, one can employ interpolation analysis. Interpolation analysis is a commonly used method for accessibility analysis. Interpolation predicts cell values in the raster on the basis of finite sample data points. Interpolation assumes that the spatially distributed objects are all spatially related. Points close to the sample points are more likely to be similar to the sample points than those farther away. GIS provides various interpolation analysis methods.

### 2.3. Cluster and Outlier Analysis

Cluster and outlier analysis can identify spatial clusters with similarly high or low values or spatial outliers with high/low values surrounded by low/high values. Anselin Local Moran’s I statistic (i.e., Local Indicator of Spatial Association, or LISA) was calculated to investigate local spatial autocorrelation in the study area. The formula is as follows [25].

(4) $$I_{i}=\frac{x_{i}-\bar{X}}{S_{i}^{2}}\sum_{j=1,j\neq 1}^{n}w_{i,j}(x_{j}-\bar{X}),\;S_{i}^{2}=\frac{\sum_{j=1,j\neq 1}^{n}{\left(x_{j}-\bar{X}\right)}^{2}}{n-1}$$

where *I_i_* is the Local Moran’s *I* statistic; *x_i_* is the spatial accessibility for the residential area *i*; n is the total number of residential areas; and *w_i,j_* is the spatial weight between residential area *i* and *j*. With a small enough *p*-value (e.g., smaller than 0.05), a positive value of *I* indicates that the residential area is surrounded by residential areas with similarly high or low spatial accessibility and thus is part of a cluster. Otherwise, the residential area is an outlier.

### 2.4. Getis-Ord Gi* Analysis

Getis-Ord G_i_* analysis (hot spot analysis) can also identify the spatial differentiation of the overall accessibility of medical facilities within the study area [26], the results of which can supplement that of cluster and outlier analysis [27]. Comparisons can be made accordingly. GIS provides the hot spot analysis tool to calculate Getis-Ord G_i_* statistics for each element in the dataset to identify spatial clusters of statistically significant hot spots (with high values of G_i_*) and cold spots (with low values of G_i_*). It also presents the locations where hot and cold spot elements cluster in space. An element should have a high (low) value and be surrounded by other elements that also have high (low) values to be a hot (cold) spot. The formula is as follows:

(5) $$G_{i}^{*}=\frac{\sum_{j=1}^{n}w_{i,j}x_{j}-\bar{X}\sum_{j=1}^{n}w_{i,j}}{S\sqrt{\frac{[n\sum_{j=1}^{n}w_{i,j}^{2}-(\sum_{j=1}^{n}w_{i,j}{)}^{2}]}{n-1}}},\;\bar{X}=\frac{\sum_{j=1}^{n}x_{j}}{n},\;S=\sqrt{\frac{\sum_{j=1}^{n}x_{j}{}^{2}}{n}-{\left(\bar{X}\right)}^{2}}$$

where G_i_* is the Getis-Ord local statistic that is a z-score; *x_j_* is the spatial accessibility for residential area *j*; *n* is the total number of residential areas; and *w_i,j_* is the spatial weight between residential area *i* and *j*.

## 3. Study Area and Data Sources

### 3.1. Study Area

**Figure 2:**
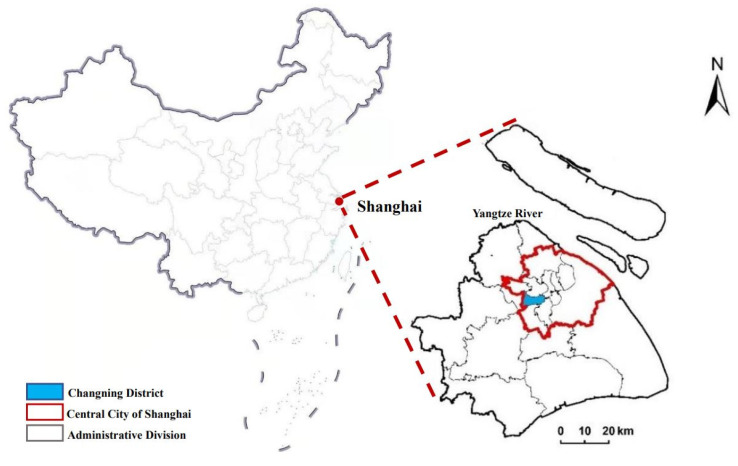
Location of Changning District in Shanghai, China.

As a megacity, Shanghai is one of the fastest-growing cities in China (see Figure 2 for the location of Shanghai). Its population comprises 24.28 million people and has a high density of almost 4000 people per square kilometer (Shanghai Statistical Yearbook, 2019). The elderly are critical users of medical facilities. Shanghai began to enter an aging society in 1979. In accordance with the standards set by the UN, an aging society refers to a region where the number of people whose ages are over 60 years has reached 10% of the total population. It was 20 years earlier than the national average, making it the first city to become an aging society in China. The number of the local elderly population (60 years old and above) in Shanghai had reached 5.18 million (35.2% of the local population) by the end of 2019. Shanghai’s level of medical treatment has been in the lead in China, with 46 tertiary hospitals (tertiary hospitals should contain more than 500 beds and take care of difficult diseases and corresponding public health functions), 101 secondary hospitals (secondary hospitals usually contain 100–499 beds and provide common disease clinics, emergency services, critical care, surgery, and inpatient services on a regional basis), and 241 primary hospitals (primary hospitals (primary healthcare institutions) usually contain 20–99 beds and mainly undertake diagnoses and treatments of frequently occurring diseases and basic healthcare). The rational distribution of medical facilities is critical in such a city with a large and aging population. However, almost all tertiary and secondary hospitals are in the city center [28].

**Figure 3:**
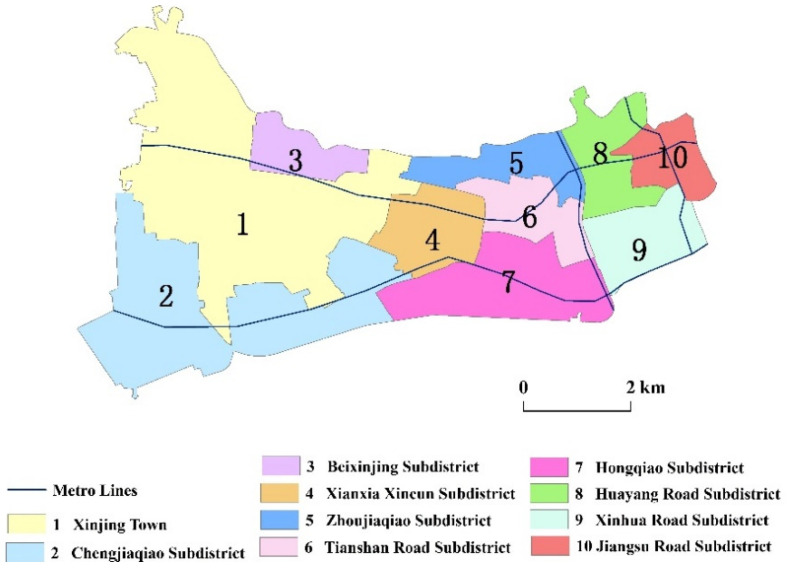
Overview of the study area.

In this study, we selected the Changning District of Shanghai as the study area. Compared with other districts in the city center, the medical facilities in Changning District are not that modern and complete (see Table 1 for details). It is a bridgehead linking Shanghai and the Yangtze River Delta, with a favorable geographic location and convenient traffic. It is on the west side of the central city of Shanghai (Figure 2). It also borders Jing’an District, Minhang District, Xuhui District, and Putuo District, and comprises 10 subdistricts (i.e., Jiangsu Road subdistrict, Huayang Road subdistrict, Xinhua Road subdistrict, Tianshan Road subdistrict, Zhoujiaqiao subdistrict, Hongqiao subdistrict, Xianxia Xincun subdistrict, Changjiaqiao subdistrict, Beixinjing subdistrict, and Xinjing Town) and 181 communities (Figure 3). The Development and Reform Commission of Changning District released the first regional indicator system in July 2017 and planned to build Changning District into an international community of excellence by 2021. One indicator was to improve the accessibility of public service facilities (including medical facilities) at the community level.

**Figure 4:**
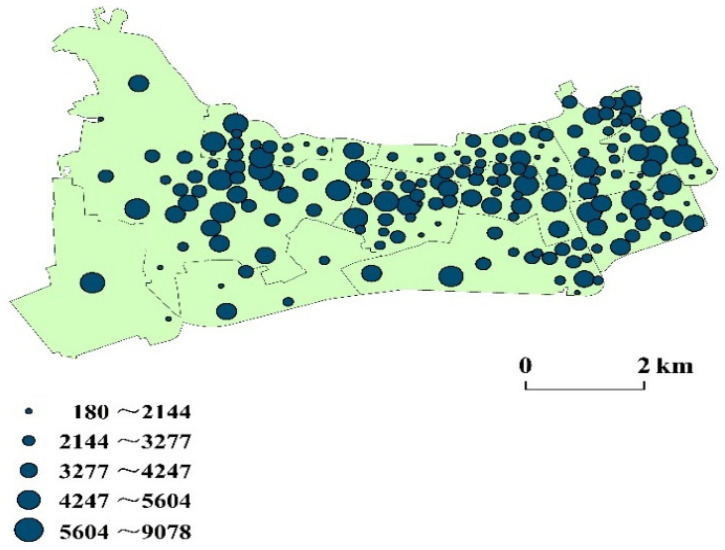
Distribution of residential areas in Changning District.

**Figure 5:**
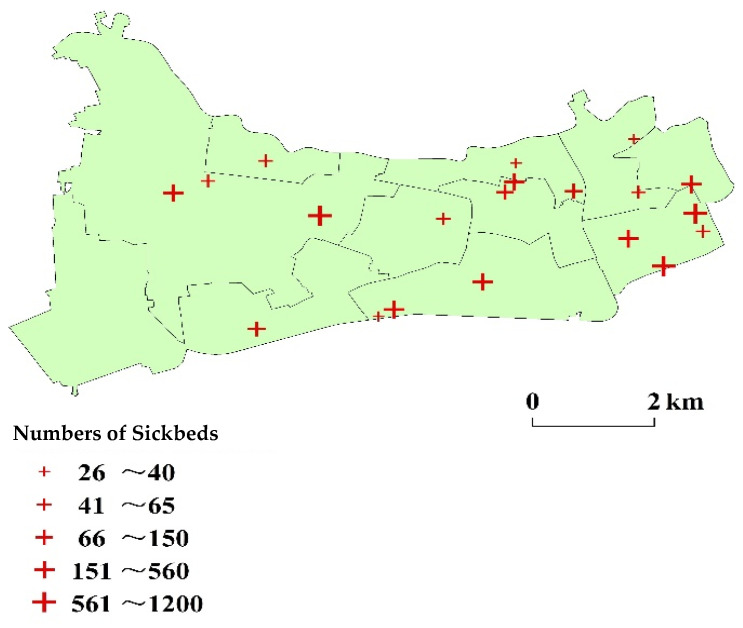
Distribution of medical facilities in Changning District.

Changning District has a population of 0.69 million and a high density of 18,120 capita per square kilometer (Shanghai Statistical Yearbook, 2019). The district comprises 218,800 elderly (60 years old and above), accounting for 38% of the local population. Overall, the population of Changning District is mainly in the north and east (for instance, Xinjing Town has the largest area and the largest population, with 146,776 people. Chengjiaqiao Street subdistrict is located in the southwest corner and has the smallest population, with only 24,487 people). The number of residents is also quite different among the 180 communities, varying from 180 to 9078 (Shanghai Sixth Population Census, 2010; Figure 4). The distribution of medical facilities in Changning District is roughly consistent with the population distribution, mainly in the north and east. Changning District has 20 public hospitals, including 1 tertiary hospital, 9 secondary hospitals, and 10 primary hospitals (Shanghai Health Planning Commission; Figure 5). The only tertiary hospital in Changning District is in its southeast. It serves as a medical hub that provides services to multiple regions.

### 3.2. Traffic Data

#### 3.2.1. Traffic Speed

The Network Analysis Module of ArcGIS calculates travel time based on the shortest route from a population point to a medical facility, considering the road network and public transport network. Shanghai’s underground track mileage ranks first in China, and the transfer rate of ground transportation and rail transit is as high as 67%, ranking second in the country. The transfer of the transportation network has become an indispensable means of transportation for residents to travel. Thus, considering the combination of ground transportation and rail transit is necessary when calculating the travel time.

For ground transportation, some studies have calculated the transit time in accordance with the vehicle speed limits of different level roads specified in Technical Standard of Highway Engineering (JTGB01-2003) and Code for Design of Urban Road Engineering (CJJ37-2012) [29,30]. However, the actual speed is different from the above norms when the ground traffic is congested. This study approximated the travel time by using the average speed of traffic flow of different level roads at peak hours. The data were from the Shanghai Comprehensive Traffic Operation Annual Report (2016) issued by the Shanghai Institute of Urban and Rural Construction and Transportation Development.

Unlike private cars and other modes of personal transport, public transport has predefined routes and schedules and is subject to frequent alteration. For rail transit, the design speed of the subway is usually 120–180 km/h. However, the operating speed of the subway is generally no more than 80 km/h given the constraining factors, such as line conditions (curves and ramps), station settings (station spacing), and dwell time (affected by the volume of passenger flow) [31]. We calculated the operating speed of each subway line (i.e., Lines 2, 3, 4, 10, and 11 in Changning District) (Lines 3 and 4 share the route in Changning District) by dividing the mileage by running time, as shown in Table 2. This method is in line with that of Zhong, Chen, and Yang [14]. The data were from the Shanghai Surveying and Mapping Institute and Shanghai Metro (Shanghai Metro Website: http://service.shmetro.com/ accessed on 15 April 2021).

#### 3.2.2. Threshold Time of Traveling to Three-Tier Hospitals

In general, residents are willing to spend a threshold time (i.e., *D_j_* in the Improved Potential Model) when traveling to a medical facility. We followed the vision of the “1560 medical circle” (“1560 medical circle”: Residents can reach primary medical care institutions within 15 min on foot and tertiary hospitals within 60 min by public transport) proposed by the Shanghai Municipal Bureau of Health and the “15 min community-life circle” (“15 min community-life circle”: residents can obtain access to public space and public facilities that meet basic needs (in terms of education, healthcare, shopping, etc.) in 15 min on foot) proposed by Shanghai Planning Guidelines of 15-Minute Community-Life Circle and Shanghai City Master Plan (2016–2040). We set the threshold time to reach primary hospitals (primary healthcare institutions) as 15 min.

We determined the threshold time of secondary hospitals through interviews. The interviews involved 150 households of 30 communities in 10 streets. Among them, 35.6% believed that traveling to secondary hospitals within 20 min is reasonable, and 50.4% pointed out that the threshold time should be no more than 30 min. Based on the interviews, we set *D_j_* of secondary hospitals as 30 min. Considering the radiation to residents of surrounding areas and cities, we set *D_j_* of tertiary hospitals to be +∞.

## 4. Results

### 4.1. Spatial Accessibility of Medical Facilities

#### 4.1.1. Determination of Friction Coefficient

The ideal method of determining the friction coefficient *β* follows the actual use of the facility. It considers the number of users at different distances from the facility [32]. However, this method often requires a large amount of data with high costs and is difficult to implement. Hence, prior studies widely used scenario analysis [19]. According to the literature, the value of parameter *β* is between 0.9 and 2.29 [33], and the common practice is to set *β* as 1.0, 1.5, 1.8, or 2.0 [34,35,36].

We calculated the spatial accessibility of medical facilities in Changning District after assigning friction coefficient *β* with values of 1.0, 1.5, 1.8, and 2.0 (Table 3). As the value of *β* increased, the maximum value of accessibility increased. The minimum value of accessibility decreased. Then, the standard deviation of accessibility increased. It indicated that the calculation results of spatial accessibility were susceptible to the value of *β*. An appropriate value of *β* needed to be chosen. By investigating the average daily travel time of residents, it was found that when *β* = 1.5, the calculated travel time was more consistent with the actual travel time. Therefore, we took *β* as 1.5 for the accessibility calculation.

#### 4.1.2. Interpolation Method Selection

To analyze the spatial accessibility of medical facilities surrounding the measurement points, we employed interpolation analysis. We compared the calculation results of the conventional interpolation method and the geostatistical method (Table 4). In general, the smaller the values of MEAN and RMSE, the more suitable the interpolation method. On this basis, the geostatistical method was superior to the conventional method in this study.

MEAN, root mean squared error (RMSE), average standard error (ASE), mean standard error (MSE), and root mean square standard error (RMSSE) are widely used indicators in evaluating prediction accuracy in geostatistics. The optimal model should meet the following criteria: (1) the absolute value of MEAN is close to 0, (2) the RMSE is the smallest, (3) the ASE is closest to RMSE, and (4) the RMSSE is close to 1. The results of the generalized cross-validation showed that Bayeskrim interpolation was relatively good (Table 5).

#### 4.1.3. Overall Spatial Accessibility of Medical Facilities

**Figure 6:**
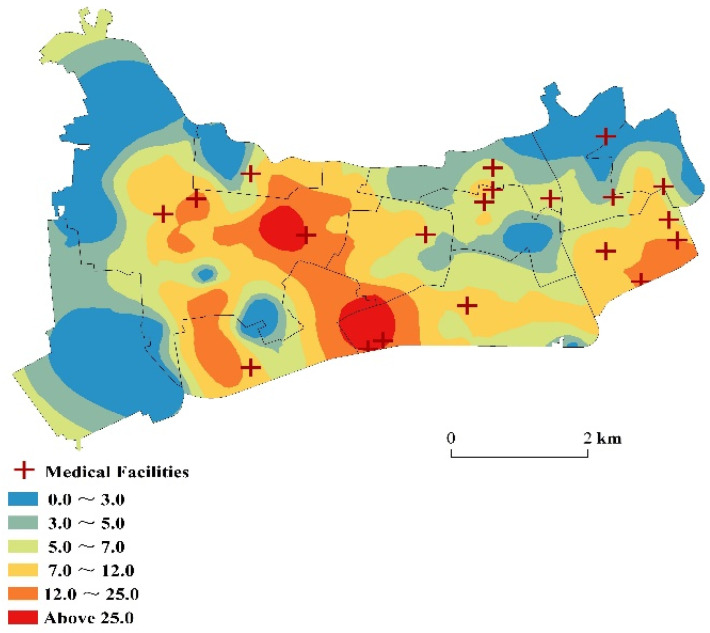
Accessibility distribution of medical facilities in Changning District.

The interpolation analysis results showed that the allocation of medical resources was imbalanced within the district (Figure 6). The central region offers convenience to those who seek medical services due to the large scale and quantity of medical facilities. The dark-colored high-value areas were mainly in Xinhua Road subdistrict and Hongqiao Street subdistrict. These three regions had obvious medical advantages. Xinhua Road subdistrict has one tertiary hospital, two secondary hospitals, and one primary hospital. These facilities form a relatively complete provision of hierarchical medical treatment. Hongqiao Street subdistrict has a secondary hospital and a primary hospital providing efficient and high-quality medical services. A complete hierarchical system of medical facilities could satisfy the needs of surrounding residents and improve the overall spatial accessibility of medical services to the neighborhood.

Five lines (i.e., Lines 2, 3, 4, 10, and 11) pass through Changning District and form a “C” shape, linking all three-tier hospitals within the area. The interpolation analysis results showed that the residential areas along the subway had better access to medical facilities than those farther away. It is because the combination of rail transportation and ground transportation can save travel time, and residents along the subway can reach medical facilities more conveniently than residents far away.

For Xinjing Town subdistrict and Chengjiaqiao subdistrict in the west of Changning District, and Huayang Road subdistrict in the northeast, there are few medical facilities and no subway lines that pass through. Residents can only rely on ground transportation to go to medical facilities far away for medical treatment, making this area less accessible to the medical facility. Supplementing medical resources and improving the transportation network will be an important way to improve the accessibility of medical treatment in this region.

#### 4.1.4. Spatial Differentiation of Medical Facilities

(1) Cluster and outlier analysis

**Figure 7:**
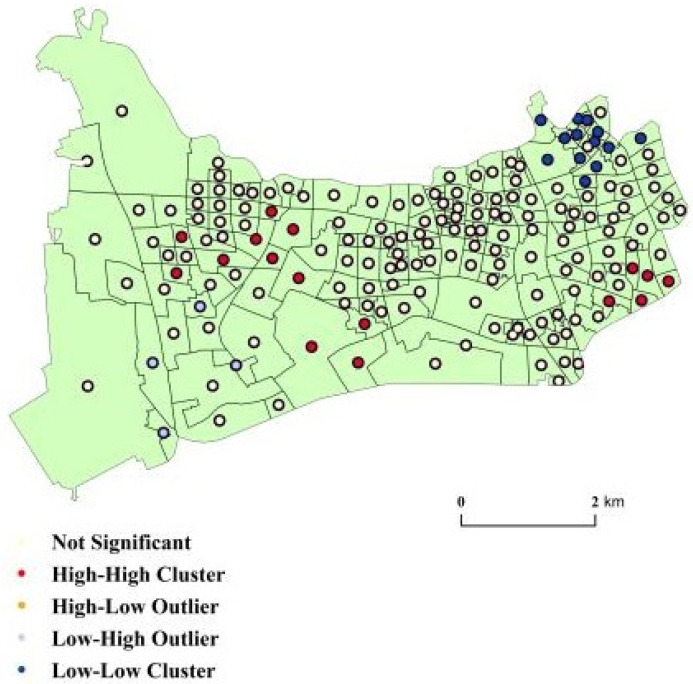
Cluster types of accessibility of medical facilities in Changning District.

To identify disparities of spatial accessibility in the study area, cluster and outlier analysis was employed. Anselin Moran’s I statistic (LISA) was calculated. Significant spatial clusters and outliers were obtained, as shown in Figure 7. High–High clusters were observed in 8.8% of the communities, and Low–Low clusters were observed in 7.2% of the communities (Table 6). Namely, 8.8% (7.2%) of the communities had high (low) spatial accessibility to medical facilities, and the same was true for their neighboring communities. The results were at the 95% confidence level. On the other hand, the spatial accessibility of most communities (81.8%) was unclustered.

**Figure 8:**
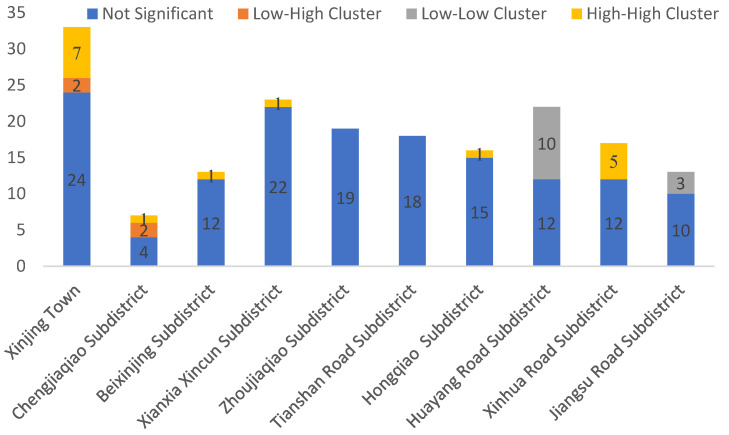
Number of communities with different types of clustering.

As shown in Figure 8, the High-High Clusters concentrated in Xinjing Town (7), Xinhua Road subdistrict (5), Chengjiaqiao subdistrict (1), Beixinjing subdistrict (1), Xianxia Xincun subdistrict (1), and Hongqiao subdistrict (1). These subdistricts have relatively high spatial accessibility of medical facilities. The Low–Low clusters were found in the two subdistricts of Huayang Road and Jiangsu Road. The spatial accessibility of medical facilities in these two districts needs improvement.

(2) Getis-Ord G_i_* analysis

**Figure 9:**
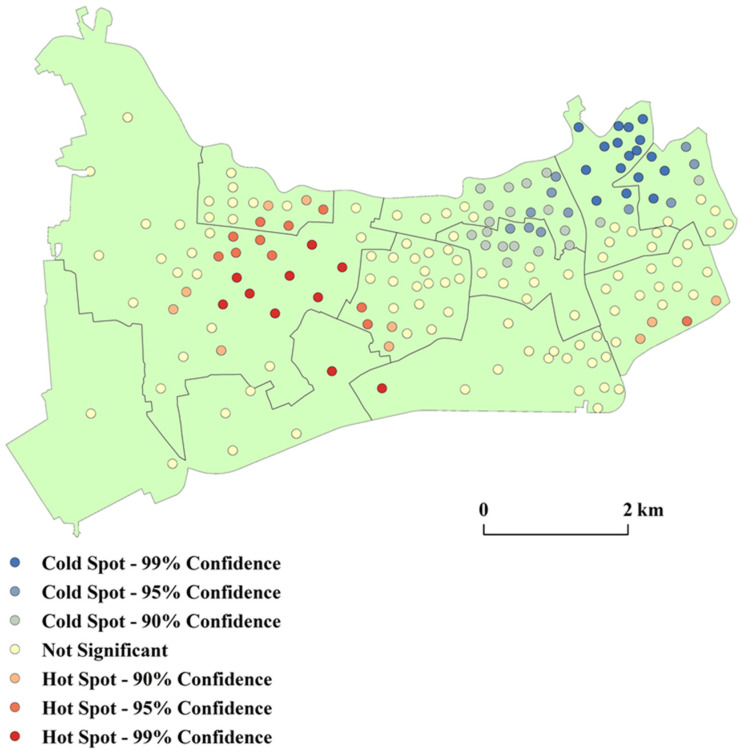
Spatial differentiation of medical facilities in Changning District.

We used Getis-Ord G_i_* analysis to depict the spatial differentiation of the medical facilities in Changning District (Figure 9). The hotspots were mainly in Xinjing Town, Beixinjing subdistrict, Xinhua Road subdistrict, and Xianxia Xincun subdistrict. They have high levels of medical services. The cold spots were mainly in the Huayang Road subdistrict, Zhoujiaqiao subdistrict, Tianshan Road subdistrict, and Jiangsu Road subdistrict. The level of medical services in these areas needs improvement. The results were consistent with that of cluster and outlier analysis.

The rest of the area belonged to the transition region, where the level of medical services also needs improvement, although not as urgent as the subdistricts with cold spots.

### 4.2. Spatial Accessibility to Different-Grade Medical Facilities

To show the relative level of spatial accessibility of different-grade medical facilities in Changning District, we calculated the ratios of the spatial accessibility from each residential area to the nearby medical facilities of all levels to its average value. Then, we divided them into five groups (Table 7). The accessibility of primary hospitals to each community was unbalanced. The spatial accessibility of secondary and tertiary hospitals was more balanced than in primary hospitals in the region. Residents generally had access to high-grade hospitals in Changning District. Among the nearby communities, 70% had poor or fair accessibility (i.e., below the average level) to primary hospitals. The proportion of communities with excellent accessibility to primary hospitals was 16.11%.

**Figure 10:**
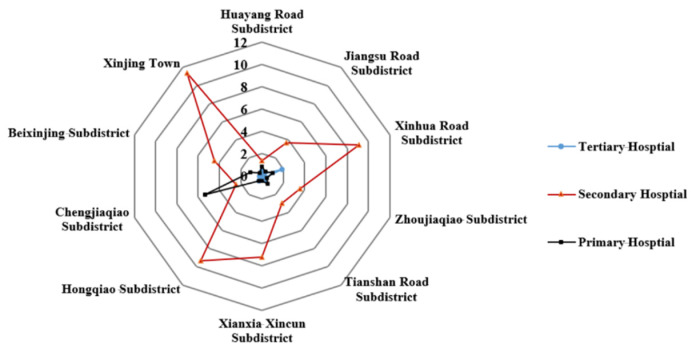
Spatial accessibility of different-grade hospitals in each subdistrict.

The above analysis examined the accessibility of different-grade medical facilities in Changning District. However, the accessibility of different-grade medical facilities in each subdistrict was unknown. Thus, we should also conduct subdistrict-level analyses. The spatial accessibility of secondary hospitals was generally better than that of tertiary and primary hospitals in the region, as shown in Figure 10. Among all subdistricts in the area, Xinjing Town, HongqiaoRoad subdistrict, Xinhua Roadsubdistrict, and XianxiaXincunsubdistrict had the best accessibility to secondary hospitals. Although the threshold travel time to tertiary hospitals was much longer than to secondary and primary hospitals, it was still unable to compensate for the disadvantages with the limited number of tertiary hospitals (only one). Xinhua Road had better accessibility to the tertiary hospital than other subdistricts. The accessibility of primary hospitals was poor in almost all subdistricts, except for Chengjiaqiao subdistrict.

## 5. Discussion

The innovations of this study are three-fold, which could supplement the current studies to a large extent. The three innovations are (1) considering the comprehensive transportation network composed of ground transportation and rail transit due to complex transportation routes, (2) calculating the spatial accessibility of small research units (i.e., communities) to more accurately reflect the rationality of allocating medical resources, and (3) considering the influence of facility levels on the residents’ medical choice behavior.

After conducting interpolation analysis, cluster and outlier analysis, and Getis-Ord G_i_* analysis, several findings are worth noting. Suggestions are put forward accordingly. First, the allocation of medical resources was imbalanced within the study area. To achieve the overall supply and demand balance of medical services, medical services in areas with high accessibility should be channeled and extended to areas with low accessibility. The development of public transportation should be accelerated in underserved areas. In China, it has become a consensus to give priority to the development of public transportation. It could significantly increase the accessibility of medical facilities by optimizing public transportation transfer, strengthening the system connection of different public transportation modes, and eliminating blind areas of medical facilities through public transportation.

Second, it was found that the accessibility of primary hospitals was poor in almost all subdistricts, except for the Chengjiaqiao subdistrict. The spatial accessibility of secondary and tertiary hospitals was more balanced than in primary hospitals. The spatial accessibility of secondary hospitals was generally better than that of tertiary and primary hospitals in the region. Constructing new branches of tertiary hospitals could improve the spatial accessibility of tertiary hospitals. However, it is difficult to set up tertiary hospitals due to the strict standards and complex procedures. Hence, policymakers should increase the number of secondary and primary hospitals and form a medical complex composed of tertiary hospitals, secondary hospitals, and primary hospitals in the region. By integrating medical resources and promoting the extension of high-quality resources of tertiary hospitals to secondary and primary hospitals, the convenience of residents’ medical treatment can be improved.

Third, to achieve the overall supply and demand balance of medical services, the efficiency of the referral system should be improved. Hospitals of different levels have different scales, technical levels, and service capabilities. Their attractiveness to residents varies. Patients usually have higher preferences for high-level hospitals. An efficient referral system could guide the hospital preferences of patients, thus affecting the spatial accessibility of medical facilities [35]. Since patients would be encouraged to go to the primary hospital first, the accessibility of primary hospitals in the study area should be improved.

This study focused on physical accessibility derived from the locations of residences and facilities. It did not investigate the driving factors underlying the spatial accessibility of different-grade hospitals, which would be looked into in future studies. Factors affecting spatial accessibility of public facilities include socio-demographic characteristics (e.g., age, gender, marital status, and education), economic factors (e.g., income and health insurance), transport infrastructure, and challenges imposed by physical conditions (e.g., terrain), etc. For instance, education, marital status, and health insurance were found to significantly affect healthcare accessibility [37]. Certain age groups (e.g., young people, the elderly) have particular travel behaviors and preferences for public facilities, thus affecting spatial accessibility [12,38]. Further, road density was found to positively affect spatial accessibility [12].

## 6. Conclusions

The rational layout of medical facilities plays an indispensable role in promoting social equity and protecting people’s lives. The accessibility of medical facilities affects access to medical services and reflects the fairness of medical facilities. This study used Changning District in Shanghai as the case study to examine the spatial accessibility of medical facilities. We introduced population scales and grades of medical facilities to the potential model. We employed community-level data and considered the travel modes of rail transit and ground traffic. The findings could facilitate identifying underserved areas with poor access to medical facilities of different levels and provide suggestions on optimizing the distribution of relevant medical facilities.

The results showed that the central area of Changning District has relatively good access to medical facilities. By contrast, the accessibility to medical facilities in the western and northeastern parts was rather weak. Regarding the accessibility of different-grade medical facilities in the region, secondary hospitals generally had better spatial accessibility than tertiary and primary hospitals. Residential areas in Xinjing Town, Hongqiao subdistrict, Xianxia Xincun subdistrict, and Xinhua Road subdistrict had the best accessibility to secondary hospitals. Xinhua Road subdistrict had relatively good accessibility to the tertiary hospital.

Changning District is in the central area of Shanghai with a dense and aging population. The rational distribution of medical facilities is critical. The proposed methodology can guide future works focusing on accessibility-related issues in other cities in China and other regions in the world. The results can facilitate local governments’ decision-making in planning public facilities (not limited to medical facilities). To ensure that residents have equitable access to medical services, policymakers and urban planners should pay special attention to the (1) equitable distribution of medical facilities, (2) efficient referral system, and (3) improvement of transport infrastructures.

As has been elaborated in Section 5, this study focused on physical accessibility derived from the locations of residences and facilities. It did not investigate the driving factors underlying spatial accessibility of medical facilities, nor did it address the access barriers of economic, social, or cultural aspects. The demographic characteristics of residents were not included in the analysis due to the data availability. Future works could look into factors affecting spatial accessibility of medical facilities (e.g., age composition, development of transport infrastructure, and road density) and use different time thresholds to perform sensitivity analysis on the spatial accessibility of medical facilities.

## Figures and Tables

**Table 1:** Distribution of three-tier hospitals in the central area of Shanghai.

District	Tertiary Hospital	Secondary Hospital	Primary Hospital	Population (1000 Persons)
Huangpu District	7	11	10	654
Changning District	1	9	10	694
Xuhui District	8	6	13	1084
Jing’an District	7	14	15	1063
Putuo District	3	6	11	1282
Hongkou District	3	7	8	797
Yangpu District	5	7	12	1313

Source: Shanghai Municipal Health Commission and Shanghai Statistical Yearbook.

**Table 2:** Speed of subway lines in Changning District.

Line	Length (km)	Number of Stations	Block Time (min)	Average Speed (km/h)
Line 2	37	22	59	37.63
Lines 3/4	-	-	-	35.65
Line 10	30.5	28	64	28.59
Line 11	56.9	31	89	38.36

**Table 3:** Spatial accessibility of medical facilities with different values of friction coefficient.

**Spatial Accessibility**	*β* = 1	*β* = 1.5	*β* = 1.8	*β* = 2
**Maximum Value**	37.89	49.15	52.06	54.17
**Minimum Value**	0.21	0.12	0.08	0.06
**Standard Deviation**	6.65	7.27	7.68	7.94

**Table 4:** Comparison of different interpolation methods.

Interpolation Method		Parameter Model	MEAN	RMSE
Conventional Interpolation Methods	IDW	γ=2.7	0.0507	6.3244
	Global polynomial interpolation	γ=3.0	0.0039	6.6480
	Local polynomial interpolation	γ=1.0	0.0884	6.5244
	RBF	Multiquadric function	0.0530	6.1926
Geostatistical Method	Empirical Bayesian Kriging	None	0.0737	6.1031
		Empirical	0.0873	6.0552
		Log empirical	0.0537	5.9836

**Table 5:** Comparison of different empirical Kriging interpolation methods.

Generalized Cross-Validation	Empirical Bayesian Kriging		
	None	Empirical	Log Empirical
*MEAN*	0.0737	0.0873	0.0537
*RMSE*	6.1031	6.0552	5.9836
*MSE*	0.0103	−0.0165	−0.0417
*RMSSE*	1.0469	1.0910	1.2041
*ASE*	5.4244	5.8663	6.4193

**Table 6:** Localized clusters of spatial accessibility based on LISA statistics.

Cluster Type	Number of Communities (%)
Unclustered	148 (81.8)
High–High	16 (8.8)
Low–Low	13 (7.2)
High–Low	0 (0)
Low–High	4 (2.2)
Total	181 (100)

**Table 7:** Levels of spatial accessibility of different-grade medical facilities in Changning District.

Level of Spatial Accessibility	Poor (<0.5)	Fair (0.5–0.75)	Average (0.75–1.0)	Good (1.0–1.25)	Excellent (>1.25)
Tertiary hospital	37.22%	35.00%	7.78%	4.44%	15.56%
Secondary hospitals	35.56%	18.89%	11.67%	6.11%	27.77%
Primary hospitals	62.78%	7.22%	10.56%	3.33%	16.11%

## Data Availability

All data analyzed during this study are indicated in the article.
